# Oculo-Cardiac Reflex

**Published:** 1916-03

**Authors:** 


**Oculo-Cardiac Reflex.** Levine, Arch. of Int. Med., May 1915, states that the slowing of a pulse from vagus inhibition, is generally absent in tabes, present in pneumonia, syphilis and chronic valvular disease. Right ocular pressure has a slightly greater effect than left. Left sided pressure has more effect on the conducting mechanism of the heart, may lengthen the time, cause partial heart block and possibly result in automatic ventricular rhythm. The phenomenon differs in different individuals and in the same individual at different times. (Note: It has seemed to the editor that vagus reflex, that is to say a slowing of the pulse by 8-10 beats a minute, was rather characteristic of what may be termed sthenic conditions, e.g. hyperchlorhydria while in asthenic conditions, there is no change or a variation of only two or three beats which can scarcely be considered significant. The opposite condition, increase in pulse rate by preponderance of the sympathetic, has not been personally observed. Some observations have been made which seem to indicate that a decided retardation of the pulse predicts a rapid 'taking hold'' by digitalis. For instance, pressure on the eye reduced the pulse in a case of hyperchlorhydria from 120 to 90. After digitalis for three days, the pulse was reduced to 70, being further reduced to 60 by ocular pressure. On the other hand, in an old man with pneumonia seen at a hospital, ocular pressure had no decided effect on the pulse. 5 drop doses of fluid extract, 4 times daily were maintained for ten days before the pulse was reduced from 140-120 to about 80. We should be pleased to receive reports on this suggestion. Ed.)

